# Quality of sweat test (ST) based on the proportion of sweat sodium (Na) and sweat chloride (Cl) as diagnostic parameter of cystic fibrosis: are we on the right way?

**DOI:** 10.1186/s13000-016-0555-6

**Published:** 2016-10-26

**Authors:** Alethéa Guimarães Faria, Fernando Augusto Lima Marson, Carla Cristina de Souza Gomez, Maria Ângela Gonçalves de Oliveira Ribeiro, Lucas Brioschi Morais, Maria de Fátima Servidoni, Carmen Sílvia Bertuzzo, Eulália Sakano, Maura Goto, Ilma Aparecida Paschoal, Mônica Corso Pereira, Gabriel Hessel, Carlos Emílio Levy, Adyléia Aparecida Dalbo Contrera Toro, Andressa Oliveira Peixoto, Maria Cristina Ribeiro Simões, Elizete Aparecida Lomazi, Roberto José Negrão Nogueira, Antônio Fernando Ribeiro, José Dirceu Ribeiro

**Affiliations:** 1 Department of Pediatrics, Faculty of Medical Sciences, University of Campinas, Campinas, Brazil; 2 Laboratory of Pulmonary Physiology, Center for Pediatrics Investigation, Faculty of Medical Sciences, University of Campinas, Campinas, Brazil; 3 Department of Medical Genetics, Faculty of Medical Sciences, University of Campinas, Campinas, Brazil; 4 Department of Otorhinolaryngology, Faculty of Medical Sciences, University of Campinas, Campinas, Brazil; 5 Department of Clinical Medicine, Faculty of Medical Sciences, University of Campinas, Campinas, Brazil; 6 Department of Clinical Pathology, Faculty of Medical Sciences, University of Campinas, Campinas, Brazil; 7 Departamento de Pediatria, Faculdade de Ciências Médicas, Universidade Estadual de Campinas, Tessália Vieira de Camargo, 126, Barão Geraldo, Cidade Universitária Zeferino Vaz, CEP: 13083-887 Campinas, São Paulo Brazil

**Keywords:** Cystic fibrosis, Diagnosis, Electrolytes, Sweat

## Abstract

**Background:**

To assess the quality of sweat test (ST) based on the proportion of sweat sodium and sweat chloride as diagnostic parameter of cystic fibrosis (CF).

**Methods:**

A retrospective study of 5,721 sweat samples and subsequent descriptive analysis were carried out. The test was considered “of good quality” (correct) when: (i) sweat chloride was lower than 60 mEq/L, and sweat sodium was higher than sweat chloride; (ii) sweat chloride was higher than 60 mEq/L, and sweat sodium was lower than sweat chloride.

**Results:**

The study included 5,692/5,721 sweat samples of ST which had been requested due to clinical presentations compatible with CF and/or neonatal screenings with altered immunoreactive trypsinogen values. Considering the proportion of sweat sodium and sweat chloride as ST quality parameter, the test was performed correctly in 5,023/5,692 (88.2 %) sweat samples. The sweat chloride test results were grouped into four reference ranges for chloride (i) chloride < 30 mEq/L: 3,651/5,692 (64.1 %); (ii) chloride ≥ 30 mEq/L to < 40 mEq/L: 652/5,692 (11.5 %); (iii) ≥ 40 mEq/L to < 60 mEq/L: 673/5,692 (11.8 %); (iv) ≥ 60 mEq/L: 716/5,692 (12.6 %). In the comparative analysis, there was no association between ST quality and: (i) symptoms to indicate a ST [respiratory (*p* = 0.084), digestive (*p* = 0.753), nutritional (*p* = 0.824), and others (*p* = 0.136)], (ii) sweat weight (*p* = 0.416). However, there was a positive association with: (i) gender, (ii) results of ST (*p* < 0.001), (iii) chloride/sodium ratio (*p* < 0.001), (iv) subject’s age at the time of ST [grouped according to category (*p* < 0.001) and numerical order (*p* < 0.001)]. For the subset of 169 patients with CF and two *CFTR* mutations Class I, II and/or III, in comparative analysis, there was a positive association with: (i) sweat chloride/sodium ratio (*p* < 0.001), (ii) sweat chloride values (*p* = 0.047), (iii) subject’s age at the time of the ST grouped by numerical order (*p* = 0.001).

**Conclusions:**

Considering that the quality of ST can be assessed by levels of sweat sodium and sweat chloride, an increasing number of low-quality tests could be observed in our sweat samples. The quality of the test was associated with important factors, such as gender, CF diagnosis, and subjects’ age.

## Background

The sweat test (ST) is considered the gold standard for the diagnosis of cystic fibrosis (CF) [1]. Around half a century has passed since its description; however, questions remain about its reproducibility and reliability, especially in cases of borderline sweat results [2]. There are only a few quality parameters to perform ST. In addition, the role of sweat sodium as quality marker for ST is unknown.

Increased chloride values observed in ST are due to mutations in the *CFTR* gene (Cystic Fibrosis Transmembrane Conductance Regulator), which encodes a protein with the same name [3]. A conclusive diagnosis of CF can be made with the identification of two mutations in the *CFTR* gene [4, 5]. It is not always possible to conduct genetic tests and perform complete *CFTR* gene sequencing for all patients, due to high costs and/or technical limitations. And yet, ST has been widely used as a tool for the diagnosis of CF for over 50 years.

Although ST has high sensitivity and specificity, it may produce inconsistent results. Analysis of ST alone may be insufficient to diagnose CF. Therefore; additional tests should be performed, such as nasal potential difference measurement, assessment of CFTR function in rectal biopsies, and evaporimetry [6–9]. Patients with CF usually have low sodium conductance, and consequently, elevated sodium concentration in sweat. This is because the activity of the epithelial sodium channel depends on the activity of the CFTR protein [10]. In CF diagnosis, sodium has a poor discriminatory power in comparison with chloride, even with an existing correlation between their levels in sweat [1, 11, 12]. Current protocols do not recommend the use of concentrations of sweat sodium as a diagnostic parameter for CF and/or as a quality marker to perform exams [12]. Although not used for CF diagnosis, concentrations of sweat sodium are analyzed by some laboratories, and used as an internal quality control procedure, since concentrations of chloride and sodium tend to be similar [1, 2, 11]. The use of chloride/sodium ratio has been proposed in order to screen patients with CF and borderline values in the ST. However, there is no consensus on this quality parameter [2].

This study aimed to verify the quality of ST based on the levels of sweat sodium and sweat chloride measured in ST of subjects with and without CF, performed in a referral center for a period of approximately 30 years with the same sweat dosage method.

## Methods

**Fig. 1:**
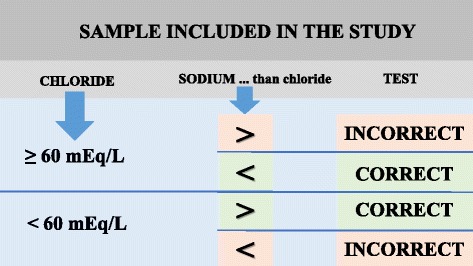
Sweat test quality by the chloride sweat and sodium sweat (proposed criteria)

A retrospective study of 5,721 sweat samples and ST descriptive analysis were carried out. The ST was considered “of good quality” (correct) when: (i) sweat chloride was lower than 60 mEq/L, and sweat sodium was higher than sweat chloride in subjects without CF; (ii) sweat chloride was higher than 60 mEq/L, and sweat sodium was lower than sweat chloride in patients with CF (Fig. 1).

The concentrations of chloride and sodium in sweat were determined by chloridrometry and flame photometry, respectively. The collection of sweat was performed by the classical Gibson & Cooke method [13].

The study was approved by the Ethics Committee from University of Campinas (Unicamp) (# 474326). The variables were collected from records of ST performed in the laboratories of the center for Gastroenterology Services and Pediatric Gastroenterology at the University Hospital of the Unicamp.

The patients’ medical records included: name, age at time of the examination, gender, medical record number, indications for ST (pulmonary, digestive, nutritional and/or others), family history of CF, weight of collected sweat sample, concentrations of sweat chloride and sweat sodium, and the chloride/sodium ratio in the sweat samples. Tests with sweat weight lower than 75 mg were excluded.

Patients were grouped into three categories according to age: (i) birth to < six months; (ii) ≥ 6 months to <18 years; (iii) ≥ 18 years [2]. The concentration of sweat chloride was used to group the sweat samples according to the CF diagnosis, as follows: (i) chloride < than 30 mEq/L; (ii) chloride ≥ 30 mEq/L to < 40 mEq/L; (iii) chloride ≥ 40 mEq/L to < 60 mEq/L; (iv) ≥ 60 mEq/L (positive test for CF) [14].

All test ordered for the same patient were analyzed, even when a patient had undergone more than one test. The study approached ST and not the result of the prevalence of CF in the samples.

For a subset of 169 patients with CF, the *CFTR* mutation screening was positive for two *CFTR* mutation Class I, II and/or III. The samples with two *CFTR* mutation Class I, II and/or III were analyzed individually. Mutations of *CFTR* were analyzed by polymerase chain reaction (PCR) (F508del) followed by enzymatic digestion (G542X, R1162X, R553X, G551D and N1303K) – [PCR/ restriction fragment length polymorphism (RFLP)]. Other mutations in the *CFTR* gene could be identified by sequencing or use of the SALSA MLPA method (Multiplex Ligation-dependent Probe Amplification) Kit P091-C1 CFTR-MRC-Holland S4X, 2183A > G, 1717-G > A, I618T with MegaBace1000^®^ (GE Healthcare Biosciences, Pittsburgh, USA) and ABI 3500 (Applied Biosystems - Thermo Fisher Scientific, São Paulo/SP, Brazil).

Descriptive statistics including numbers of observations, means, standard deviations, medians, minimums and maximums were used to summarize continuous variables. Confidence intervals (95 %) were calculated for proportions. Categorical data was presented as tables of frequency counts and associated percentages.

Statistical analysis was performed using SPSS software (Statistical Package for Social Sciences) version 22.0 (SPSS Inc., Chicago, IL, USA) [15]. The comparison between variables with categorical distribution was carried out by Test χ^2^ (Pearson and Likelihood ratio) and Fisher’s exact test, depending on the data distribution. For the analysis of variables with numerical distribution, Fisher’s exact test and one-way analysis of variance were used. Non-parametric statistical test, such as Mann-Whitney and Kruskal-Wallis, were applied when necessary. The graphics and identification of difference between the groups obtained by Kruskal-Wallis test were performed in MedCalc^®^ for Windows, version 16.1 (MedCalc^®^ Software, Ostend, Belgium). α = 0.05 was set for all analyses. The GPower software version 3.1.9.2 (Moorenstraße, Düsseldorf, Germany) [16, 17] was used to calculate the power of the sample adopting power value above 80 %.

## Results

The study included 5,721 sweat samples of ST, which had been requested due to clinical presentation compatible with CF and/or neonatal screening with altered immunoreactive trypsinogen values. Of these sweat samples, 29 (0.51 %) were excluded: (i) 23 showed sweat weight lower than 75 mg; (ii) four had no indication about sweat weight; (iii) one lacked laboratory data; (iv) one had no sodium value. Thus, 5,692 sweat samples were included in this study. The gender of 17 subjects (0.3 %) could not be determined, as the tests had been carried out with the newborn’s mother’s name, after newborn screening. So, 3,023 sweat samples collected from males (53.3 %) and 2,652 (46.7 %) sweat samples collected from females were included and analyzed.

The mean age of the subjects was 12.12 ± 17.84 years; and median 4 (ranging from 0 to 85.58) years. In 146 (2.6 %) sweat samples, there was no record to confirm the exact age at the time of the examination. The following frequency was obtained for each age group: (i) ≤ 0 to 6 months: 634/5,611 (11.33 %) samples; (ii) > 6 months to ≤ 18 years: 3,897/5,611 (69.5 %) samples; (iii) > 18 years: 1,080/5,611 (19.2 %) samples.

The mean sweat chloride concentration was 32.45 ± 27.67 mEq/L, median 22.30 (ranging from 1 to 213.10) mEq/L. The mean sweat sodium level was 36.45 ± 21.56 mEq/L, median 29.5 (ranging from 6.30 to 154.70) mEq/L. Clinical indications for having the sweat test performed included (i) breathing symptoms: 2,920/3,791 (77 %); (ii) digestive symptoms: 464/3,791 (12.2 %); (iii) nutritional symptoms: 435/3,791 (11.5 %); (iv) others: 467/3,791 (12.3 %). The initial medical request for ST of 1,901/5,692 (33.4 %) sweat samples could not be obtained. The sweat chloride/sodium ratio showed a mean level of 0.821 ± 0.250; median of 0.799 (ranging from 0.06 to 2.51).

Considering the quality of the ST based on the proportion of sweat chloride and sweat sodium, the test was performed correctly in 5,023/5,692 (88.2 %) samples, and incorrectly in 669/5,692 (11.8 %).

The sweat samples were grouped into four reference ranges for chloride and their respective interpretative comments: (i) chloride < 30 mEq/L: 3,651/5,692 (64.1 %); (ii) chloride ≥ 30 mEq/L to < 40 mEq/L: 652/5,692 (11.5 %); (iii) chloride ≥ 40 mEq/L to < 60 mEq/L: 673/5,692 (11.8 %); (iv) chloride ≥ 60 mEq/L: 716/5,692 (12.6 %).

**Fig. 2:**
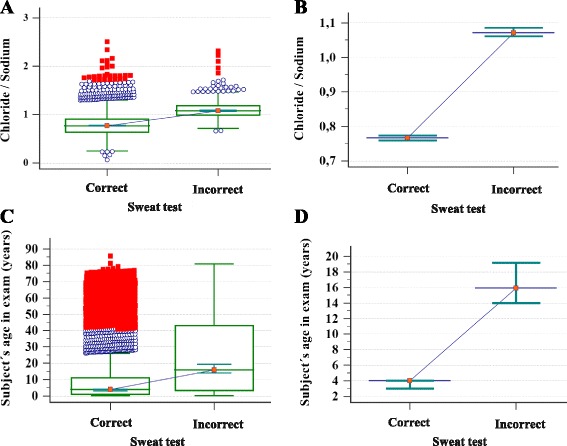
**a** and **b** Association between quality of sweat test (proposed criteria) and sweat chloride/sodium ratio. Correct: *N* = 5,023; mean = 0.784 ± 0.234; median = 0.766; range from 0.06 to 2.51; Incorrect: *N* = 669; mean = 1.102 ± 0.184; median = 1.071; range from 0.66 to 2.31. *p* < 0.001. **c** and **d** Association between quality of the sweat test and the subject’s age at the time of the examination. Correct: *N* = 4912; mean = 10.545 ± 16.523; median = 4; range from 0 to 85.58; Incorrect: *N* = 634; mean = 24.370 ± 22.366; median = 15.92; range 0 to 80.83. *p* < 0.001. Statistical analysis was performed by Mann-Whitney test. α = 0.05

In comparative analysis, there was no association between the quality of ST and: (i) symptoms to request the test [breathing (*p* = 0.084), digestive (*p* = 0.753), nutritional (*p* = 0.824) and others (*p* = 0.136)], and (ii) sweat weight (*p* = 0.416). At the same time, there was a positive association with: (i) gender (*p* = 0.001), (ii) result of ST (*p* < 0.001), (iii) sweat chloride/sodium ratio (*p* < 0.001), and (iv) subject’s age at the time of the ST [grouped by category (*p* < 0.001) and numerical order (*p* < 0.001)] (Table 1 and Fig. 2).

**Table 1:** Comparison between the quality of the sweat test based on the concentrations of sweat chloride and sweat sodium (proposed criteria) and the gender and age of subjects examined, as well as the results of the sweat test in view of the sweat chloride concentration obtained in the exam

Variable	Group	Quality of Sweat Test by Proposed Criteria			*p*-value	OR^correct^	95 % CI	OR^incorrect^	95 % CI
		Correct	Incorrect	Total					
Gender	Male	2707	316	3023	0.001	1.311	1.115 to 1.541	0.763	0.649 to 0.897
	Female	2300	352	2652		1	-	1	-
	Total	5007	668	5675					
Result of diagnosis of cystic fibrosis	<30 mEq/L	3462	189	3651	< 0.001	5.633	4.711 to 6.734	0.178	0.148 to 0.212
	≥ 30 to < 40 mEq/L	538	114	652		0.584	0.468 to 0.728	1.712	1.374 to 2.135
	≥ 40 to < 60 mEq/L	471	202	673		0.239	0.198 to 0.289	4.180	3.456 to 5.057
	≥ 60 mEq/L	552	164	716		0.380	0.312 to 0.463	2.630	2.160 to 3.203
	Total	5023	669	5692					
Subject’s age	0 to 6 months	598	36	634	< 0.001	2.312	1.634 to 3.27	0.433	0.306 to 0.612
	> 6 months to ≤ 18 years	3591	306	3897		2.883	2.44 to 3.405	0.347	0.294 to 0.410
	> 18 years	778	302	1080		0.210	0.177 to 0.25	4.755	4.001 to 5.650
	Total	4967	644	5611					

*OR* odds ratio, *CI* confidence interval, *%* percentage, *mEq/L* milliequivalents per liter. Alpha = 0.05

For the subset of 169 patients with CF and two *CFTR* mutations Class I, II and/or III, in comparative analysis. There was no association between the quality of ST and: (i) gender (*p* = 1); (ii) subject’s age at the time of the ST grouped by category (*p* = 0.128); (iii) symptoms to request the test [breathing, digestive, nutritional and others – all patients with CF showed corrected data (*p* > 0.05)], (iv) sweat weight (*p* = 0.191), (v) sweat sodium values (*p* = 0.151). At the same time, there was a positive association with: (i) sweat chloride/sodium ratio (*p* < 0.001), (ii) sweat chloride values (*p* = 0.047), (iii) subject’s age at the time of the ST grouped by numerical order (*p* = 0.001) (Table 2).

**Table 2:** Comparison between the quality of the sweat test based on the concentrations of sweat chloride and sweat sodium (proposed criteria) and age of subjects examined, as well as the results of the sweat test in view of the sweat chloride concentration obtained in the exam. All subjects had cystic fibrosis and two *CFTR *mutations Class I, II and/or III

Variable	Group	Number	Mean ± SD	Median	Minimum	Maximum	*p*-value
Sweat chloride/sodium ratio	Correct	155	1.33 ± 0.24	1.27	1	2.11	<0.001
	Incorrect	4	0.94 ± 0.03	0.93	0.9	0.97	
Sweat chloride values	Correct	155	112.48 ± 19.70	111.61	63.70	159.20	0.047
	Incorrect	4	93.34 ± 13.52	94.18	79.61	105.40	
Subject’s age	Correct	133	3.47 ± 5.53	1	0	37.33	0.001
	Incorrect	2	24.13 ± 8.66	24.12	18	30.25	

*N* number of patients, *SD* standard deviation, *CFTR ﻿Cystic ﻿fibrosis transmembrane regulator*. The statistical analysis was performed by the Mann-Whitney test. Alpha = 0.05

The frequency of *CFTR* mutations Class I, II and/or III is showed in the Table 3.

**Table 3:** Distribution of patients with cystic fibrosis considering the genotype for mutations in the CFTR gene and classes of identified mutations

Genotype	Number	Percent	Group of patients
F508del/F508del	88^a^	52.1	Patients with two Class I, II and/or III
F508del/G542X	22	13	
F508del/N1303K	8^b^	4.7	
F508del/R1162X	8	4.7	
F508del/R553X	5	3	
F508del/1584-18672pbA > G	4	2.4	
F508del/c.1717-1G > A	3	1.8	
F508del/R1066C	4	2.4	
3120 + 1G > A/R1066C	3	1.8	
F508del/2183AA > G	1	0.6	
F508del/ 6b-16 exon duplication	2	1.2	
F508del/G85E	2	1.2	
F508del/S549R (T > G)	2	1.2	
F508del/S4X	3^c^	1.8	
G542X/2183AA > G	1	0.6	
G542X/R1162X	2	1.2	
R1162X/R1162X	4	2.4	
F508del/1812-1G > A	4	2.4	
2183AA > G/2183AA > G	2	1.2	
3120 + 1G > A/3120 + 1G > A	1	0.6	

^a^ 4 patients with cystic fibrosis and normal sweat chloride values were excluded (sweat chloride values: 13.10 mEq/L; 21,90 mEq/L; 35.70 mEq/L; 55.30 mEq/L); ^b^ 1 patient with cystic fibrosis and normal sweat chloride values in 5 sweat tests was excluded (sweat chloride values: 21.60 mEq/L; 23.44 mEq/L; 24.40 mEq/L; 29.50 mEq/L; 47 mEq/L); ^c^ 1 patient with cystic fibrosis and normal sweat chloride value was excluded (sweat chloride value: 52.40 mEq/L); N, Sample size; *CFTR*, *Cystic fibrosis transmembrane regulator*

## Discussion

Patients with two identified mutations in the *CFTR* gene do not usually show normal sweat values in their tests. CF patients show proportionately elevated values for both sodium and chloride electrolytes; with a difference between them, that does not usually exceed 15 mEq/L. In CF, sweat sodium concentration is usually lower than sweat chloride concentration, and the opposite relationship is observed in individuals without CF [18].

In this study, this parameter was used to assess the quality of ST. When comparing the quality of ST with gender, it was observed that the numbers of correct tests were greater in males than females. A possible explanation is the fact that women produce lower sweat volume due to the constitution of their sweat glands. Men have fewer active sweat glands, but higher sweating rate per gland. Women show lower cholinergic and β-adrenergic sweat secretion rates than men [8, 9].

The comparison between the quality and results of ST for CF diagnosis showed a higher number of incorrect tests in the chloride concentration range of 40 to 60 mEq/L, known as borderline range for the ST, as determined by Gibson and Cooke [14]. Some studies approach the need to assess the test results with age-related reference intervals [1, 2, 11, 12, 19]. Patients with clinical CF and chloride levels in ST between 30 and 59 mEq/L may have two mutations in the *CFTR* gene [12, 20].

Sweat chloride reference value between 30 and 59 mEq/L is associated with borderline range, depending on the individual’s age, and it may possibly include individuals with Transmembrane Conductance Regulator Related Metabolic Syndrome. It is estimated that 8 to 15 % of subjects in this group may receive delayed diagnosis of CF and the initial clinical presentation of CF may be confused with other respiratory diseases [21, 22].

According to the Cystic Fibrosis Foundation, CF is likely to be diagnosed when chloride concentration is greater than or equal to 60 mEq/L in two-sample ST. For infants up to six months of age, CF is very unlikely to be diagnosed when the chloride concentration is equal to or less than 29 mEq/L; as well as for individuals older than six months of age, when chloride concentration is equal to or less than 39 mEq/L. In our study, a greater number of incorrect tests were observed in the age group over 18 years. It appears that sweat chloride peaked in adults over 18 years of age, suggesting that the borderline value of 60 mEq/L to diagnose CF may not be sensitive for all age groups [2]. During the first 24 h after birth, sweat electrolyte values may be transiently elevated in normal infants, followed rapid decline of electrolytes in the first days of life. Moreover, it can be difficult to obtain adequate amount of sweat during the first weeks after birth, especially in preterm infants [23].

The concentration of electrolytes in the sweat increases with age and healthy adults may have chloride levels above 60 mEq/L [24, 25]. Furthermore, at the time of interpretation of ST, it should be considered that some rare *CFTR* gene may be related to borderline or negative values ST [18, 25].

In addition to CF, some diseases may cause increased concentrations of sweat chloride, and most diseases can be differentiated based on clinical presentations. Some examples include: atopic dermatitis, hypogammaglobulinemia, glycogen storage disease type I, mucopolysaccharidosis type I, nephrogenic diabetes insipidus, pseudohypoaldosteronism, celiac disease, adrenal insufficiency, and untreated hypothyroidism. False positive result may occur in case of malnutrition, dehydration, skin conditions (eczema or rash) and ST technical errors during induction, collection and measure of chloride and sodium concentrations [18, 25].

False negative result is related to the presence of edema, use of mineralocorticoid, collection and analysis of insufficient amount of sweat, and other technical problems [25, 26]. Sweat sample was collected by experienced personnel in accordance with international guidelines and internal quality control procedures, in order to minimize possible methodological errors and misdiagnosis, as some symptoms may resemble those of CF.

There were two limitations to this study. First, this study did not include controls: all subjects were referred to ST due to their clinical manifestations, positive newborn screening results for CF, or family history. Second, it was not possible to confirm a diagnosis of CF for all patients using a genetic study. However, the data including *CFTR* mutations was included in the present manuscript and showed similar results as the first analysis for all exams performed.

## Conclusions

It is assumed that quality of the ST can be assessed by concentrations of sweat sodium and sweat chloride; however, our study showed a great number of poor quality sweat tests. The quality of the tests was associated with some important factors, such as gender, CF diagnostic results, and age of subjects. Although ST is considered the gold standard for the diagnosis of CF, it has limitations and may produce both false positive and false negative results. Constant efforts should be targeted to understand ST results and seek quality markers to perform the tests, in order to allow accurate screening of patients. The objective is to make identification of mutations in the *CFTR* gene possible for all patients and/or to make it a regular screening method for patients suspected of having CF. The *CFTR* mutation screening can only be achieved with minimized costs and improved technical resources, which enable complete *CFTR* gene sequencing, and include all mutations with their respective classes and types. Thus, considering the proportion adopted in this study, the quantification and use of sweat sodium is still needed in ST. Special attention should be paid to borderline range for the diagnosis of CF, where a greater chance of errors could be observed.

## Abbreviations

CF: CYSTic fibrosis
CFTR: Cystic fibrosis transmembrane conductance regulator
SPSS: Statistical package for social sciences
ST: Sweat test
Unicamp: University of Campinas
