# Erratum to: Quality of sweat test (ST) based on the proportion of sweat sodium (Na) and sweat chloride (Cl) as diagnostic parameter of cystic fibrosis: are we on the right way?

**DOI:** 10.1186/s13000-017-0611-x

**Published:** 2017-02-27

**Authors:** Alethéa Guimarães Faria, Fernando Augusto Lima Marson, Carla Cristina de Souza Gomez, Maria Ângela Gonçalves de Oliveira Ribeiro, Lucas Brioschi Morais, Maria de Fátima Servidoni, Carmen Sílvia Bertuzzo, Eulália Sakano, Maura Goto, Ilma Aparecida Paschoal, Mônica Corso Pereira, Gabriel Hessel, Carlos Emílio Levy, Adyléia Aparecida Dalbo Contrera Toro, Andressa Oliveira Peixoto, Maria Cristina Ribeiro Simões, Elizete Aparecida Lomazi, Roberto José Negrão Nogueira, Antônio Fernando Ribeiro, José Dirceu Ribeiro

**Affiliations:** 1 Department of Pediatrics, Faculty of Medical Sciences, University of Campinas, Campinas, Brazil; 2 Laboratory of Pulmonary Physiology, Center for Pediatrics Investigation, Faculty of Medical Sciences, University of Campinas, Campinas, Brazil; 3 Department of Medical Genetics, Faculty of Medical Sciences, University of Campinas, Campinas, Brazil; 4 Department of Otorhinolaryngology, Faculty of Medical Sciences, University of Campinas, Campinas, Brazil; 5 Department of Clinical Medicine, Faculty of Medical Sciences, University of Campinas, Campinas, Brazil; 6 Department of Clinical Pathology, Faculty of Medical Sciences, University of Campinas, Campinas, Brazil; 7 Departamento de Pediatria, Faculdade de Ciências Médicas, Universidade Estadual de Campinas, Tessália Vieira de Camargo, 126, Barão Geraldo, Cidade Universitária Zeferino Vaz, CEP: 13083-887 Campinas, São Paulo Brazil

## Erratum

During production of the original article [1] the Methods section included an incorrect sentence. The following sentence “For the analysis of variables with numerical distribution, Fisher’s exact test and one-way analysis of variance were used” should be corrected as “For the analysis of variables with numerical distribution, Student’s t-test and one-way analysis of variance were used”.

## References

[1] Quality of sweat test (ST) based on the proportion of sweat sodium (Na) and sweat chloride (Cl) as diagnostic parameter of cystic fibrosis: are we on the right way? BMC Diagnostic Pathology. 2016;11:103. doi:10.1186/s13000-016-0555-6. 10.1186/s13000-016-0555-6 PMC5080702 27784314

